# Interactions Between Cells of *Sulfobacillus thermosulfidooxidans* and *Leptospirillum ferriphilum* During Pyrite Bioleaching

**DOI:** 10.3389/fmicb.2020.00044

**Published:** 2020-01-29

**Authors:** Qian Li, Jianyu Zhu, Shoupeng Li, Ruiyong Zhang, Tangfu Xiao, Wolfgang Sand

**Affiliations:** ^1^Key Laboratory for Water Quality and Conservation of the Pearl River Delta, Ministry of Education, School of Environmental Science and Engineering, Guangzhou University, Guangzhou, China; ^2^Key Laboratory of Biometallurgy of Ministry of Education, School of Minerals Processing and Bioengineering, Central South University, Changsha, China; ^3^School of Environmental Science and Engineering, Guangdong University of Technology, Guangzhou, China; ^4^Federal Institute for Geosciences and Natural Resources (BGR), Hanover, Germany; ^5^College of Environmental Science and Engineering, Donghua University, Shanghai, China; ^6^Biofilm Centre, Aquatische Biotechnologie, Universität Duisburg-Essen, Essen, Germany

**Keywords:** *Sulfobacillus thermosulfidooxidans*, *Leptospirillum ferriphilum*, bioleaching, adhesion, biofilm

## Abstract

*Sulfobacillus* and *Leptospirillum* occur frequently in leaching systems. Here we investigated the effects of cells of *L. ferriphilum* on biofilm formation and leaching performance by *S. thermosulfidooxidans*. The effects were caused by the presence of *L. ferriphilum* or an addition of pyrite leach liquor from *L. ferriphilum*. Data show that the number of attached *S. thermosulfidooxidans* on pyrite increases, if the pyrite had been pre-colonized by living biofilms of *L. ferriphilum*, while it decreases if the pre-colonized biofilms had been inactivated. Coaggregation between *S. thermosulfidooxidans* and *L. ferriphilum* occurs during the dual-species biofilm formation, but different effects on bioleaching were noted, if the preculture of *L. ferriphilum* had been different. If *L. ferriphilum* had been pre-colonized on a pyrite, significantly negative effect was shown. However, if the two species were simultaneously inoculated into a sterile leaching system, the bioleaching efficiency was better than that of a pure culture of *S. thermosulfidooxidans*. The effect might be related to a metabolic preference of *S. thermosulfidooxidans*. If *S. thermosulfidooxidans* performed leaching in a filtered pyrite leachate from *L. ferriphilum*, the cells preferred to oxidize RISCs instead of ferrous ion and the number of attached cells decreased compared with the control. This study gives an indication that in a short-term multi-species leaching system the role of *S. thermosufidooxidans* may be related to the time of its introduction.

## Introduction

Microorganisms catalyze metal ion leaching from insoluble metal sulfides, such as pyrite (FeS_2_), arsenopyrite (FeAsS), chalcopyrite (CuFeS_2_), sphalerite (ZnS) or marcasite (FeS_2_) by biological oxidation via contact and non-contact modes. Non-contact leaching is mainly exerted by planktonic bacteria, while contact leaching requires bacteria attached on the mineral surface. In both cases ferric ion is the most important oxidizing agent. As a consequence of the attack on metal sulfides ferrous ions and reduced inorganic sulfur compounds (RISCs) are produced. Ferrous ion is then re-oxidized to ferric ion by the iron-oxidizing microorganisms, and RISCs are oxidized to sulfate by sulfur-oxidizing bacteria. As a result, minerals get dissolved (Sand et al., 2001). This process is called bioleaching or biomining. In this way it is applied industrially to win metals from low-grade metal sulfides because of low cost and environmentally less harmful than the conventional mineral processing. However, if this process happens in a natural environment, then it may cause serious problems known as acid mine drainage (AMD) with high metal concentrations and extremely low pH (Hoffert, 1947).

Molecular analysis in the leachate of industrially or naturally leaching systems shows that microbial consortia of multiple species occur and the genus *Sulfobacillus* and *Leptospirillum* are detected frequently (Okibe et al., 2003; Johnson et al., 2008; Acosta et al., 2014). The genus *Sulfobacillus* was described by Golovacheva and Karavaiko in 1978 (Golovacheva and Karavaĭko, 1978). Sulfobacilli are Gram-positive, generally non-motile, rod shaped, endospore-forming and moderately thermophilic acidophilic bacteria. The temperature range for their growth is 17–60°C, with an optimum around 40–55°C. The pH range for their growth is 1.1–5.5, with an optimum between 1.7 and 2.4 (Bogdanova et al., 2006). They can oxidize ferrous ion, reduced RISCs such as tetrathionate, thiosulfate and elemental sulfur, as well as sulfide minerals in the presence of 0.02% yeast extract. They always are members of microbial leaching communities because of their versatile metabolism and also because of their high tolerance to low pH and high metal concentrations (Rawlings and Johnson, 2007). Decreased amounts of sulfobacilli even result in reduced bioleaching efficiency for chalcopyrite (Spolaore et al., 2011).

Markosyan isolated *Leptospirillum* in 1972 and described it as a mesophilic, vibrioid-shaped and iron-oxidizing bacterium (Markosyan, 1972). Leptospirilli are Gram-negative bacteria with a lot of remarkable properties, which allow them to thrive in leaching habitats. Compared with acidithiobacilli, leptospirilli can resist 10 times higher ferric ion concentrations than acidithiobacilli. Their optimum growth temperature and pH is 22–35°C and 1.5–2.3, respectively (Sand et al., 1992). Leptospirilli attach to metal sulfides fast and firmly (Sand et al., 1992; Zhu et al., 2012) and are pivotal for biofilm formation in mixed cultures. They are pre-colonizers helping other microorganisms attach to substrata (Noël et al., 2010).

The understanding of interactions at the strain and at the species or genus level in bioleaching communities under such extreme conditions is still in its infancy but would be very helpful to enhance bioleaching or to control AMD. Okibe et al. (Okibe and Johnson, 2004) investigated the interactions between moderately thermophilic prokaryotes during bioleaching of pyrite. They assume interactions between these acidophiles, because they found that different combinations of these acidophiles had either positive or negative effects on the dissolution of pyrite. Castro et al. (Castro et al., 2016) showed that *Sulfolobus metallicus* and *Acidianus copahuensis* negatively influenced each other during initial attachment and pyrite dissolution, and physical contact between the two species was detected. Maulani et al. (2016) found that pre-established biofilms of *Leptospirillum ferriphilum* inhibited the initial attachment to pyrite by cells of *Ferroplasma acidiphilum* and did not promote pyrite leaching by *F. acidiphilum*. In contrast, inactivated biofilm cells of *S. thermosulfidooxidans* enhanced pyrite bioleaching by *F. acidiphilum*. Bellenberg et al. (2014) reported that molecular cell-to-cell communication plays a role in regulating biofilm formation in leaching systems, and their data also indicate other still undefined interactions. Liu et al. (2016) investigated the influence of *S. thermosulfidooxidans* on the Archaeon *Acidianus* sp. DSM 29099 during leaching of pyrite. They described that leaching by *Acidianus* sp. DSM 29099 becomes enhanced with either cells or filtered leachates of *S. thermosulfidooxidans*. However, little work has been done to analyze the interactions between the genus *Leptospirillum* and *Sulfobacillus*, although both are often dominant in leaching operations.

In order to investigate such interactions in this study *S. thermosulfidooxidans* DSM9293^T^ and *L. ferriphilum* DSM 14647^T^ were chosen. Since *S. thermosulfidooxidans* DSM9293^T^ is known to be a weak biofilm former (Li et al., 2016), we used *L. ferriphilum*, either as living cells or inactivated biofilms or filtered leachates to influence biofilm formation and pyrite leaching by *S. thermosulfidooxidans*. The results show that the number of attached *S. thermosulfidooxidans* on pyrite increased, if the pyrite was pre-colonized by living biofilms of *L. ferriphilum*, while it decreased if the biofilms had been inactivated previously. *L. ferriphilum* facilitates biofilm formation by *S. thermosulfidooxidans*, if dual-species biofilms can form on pyrite. Physical contact between *S. thermosulfidooxidans* and *L. ferriphilum* illustrates that coaggregations between the two species were formed, but with different consequences. If *L. ferriphilum*, either living or inactivated, had pre-colonized pyrite, the leaching performance of *S. thermosulfidooxidans* was worse than that of either single culture. However, when the two species were simultaneously inoculated to a sterile leaching system, the leaching performance was much better than before. When *S. thermosulfidooxidans* performed leaching in filtered leachate from *L. ferriphilum*, a high percentage of ferrous ion and low pH of the system illustrate it preferred to oxidize RISCs over ferrous ion, and numbers of attached cells decreased compared with the control. The results indicate that in a multi-species leaching system the role of *S. thermosufidooxidans* might be decided by the time when it is introduced: it is more like an iron oxidizer if added initially with other species, while it prefers to be a sulfur oxidizer if it is not introduced at the beginning of the bioleaching. Our study provides information for investigating species-species interactions in a bioleaching system, which is helpful for researchers to optimize a leaching consortium or to prevent bioleaching happening in natural environment.

## Materials and Methods

### Strains and Cultivation

Strains *S. thermosulfidooxidans* DSM 9293 and *L. ferriphilum* DSM 14647 were purchased from Deutsche Sammlung von Mikroorganismen und Zellkulturen (DSMZ), Germany. The two strains were cultivated in Mackintosh (MAC) medium (Mackintosh, 1978). 0.02% yeast extract was supplemented in the medium for *S. thermosulfidooxidans*. 2% (m/v) pyrite grains or 1% sulfur prills or sulfur slices were added as energy source for *S. thermosulfidooxidans*. 4 g/L ferrous ion was added as energy source for *L. ferriphilum*. The initial pH of the medium was adjusted to 2.5 when the energy source was sulfur, while the pH was adjusted to 1.8 when the energy source was pyrite or ferrous ion. Both strains were cultivated on a rotary shaker at 140 rpm. Culturing temperature for *S. thermosulfidooxidans* and *L. ferriphilum* was 45 and 37°C, respectively.

### Preparation of Pyrite Grains, Pyrite Slices, Sulfur Prills and Sulfur Slices

All pyrite was museum grade in form of naturally crystalized cubes from Freiberg, Germany. Smooth and shiny pyrite cubes were used for the experiments. The cubes were cut by a diamond saw and slices with one shiny side and one rough side were obtained. Only the shiny side was used for biofilm formation. The remaining pyrite was ground and pyrite grains with a size of 50–100 μm were used for cultivation or bioleaching experiments. Pyrite grains and slices were cleaned and sterilized according to Schippers et al. (1996). Briefly, they were washed with 6 M boiling HCl for 30 min, then rinsed with deionized water till the pH was neutral. After washing with acetone for three times, they were dried at 80°C for 12 h and sterilized for 24 h at 120°C under a nitrogen atmosphere.

To produce sulfur prills, elemental sulfur powder was firstly molten in a glass beaker at 130°C and then poured into cold deionized water with an agitation of 250 rpm. Due to rapid cooling sulfur prills with a diameter of 2 mm were formed. To produce sulfur slices, molten sulfur was poured onto glass plates to obtain a sulfur layer after its solidification. Then sulfur slices with a size of 3 cm × 3 cm × 0.2 cm were obtained by manually cutting the sulfur layer. Both sulfur prills and slices were autoclaved at 110°C for 90 min.

### Bioleaching of Pyrite by *S. thermosulfidooxidans* in the Presence of *L. ferriphilum*

Pyrite grains were first incubated at a pulp density of 2% (m/v) with 10^8^ cells/mL of iron-grown *L. ferriphilum* in 50 mL MAC medium with a pH of 1.8 for 1 day on a rotary shaker at 37°C. Afterward, the culture supernatants were discarded and the pyrite residues were washed three times with fresh MAC medium. After washing the pyrite residues were processed as following: Resuspended the pyrite residues in 50 mL fresh medium; Resuspended the pyrite residues in 50 mL fresh medium which contained 0.02% yeast extracts and 10^8^ cells/mL of sulfur-grown cells of *S. thermosulfidooxidans*; Incubated the pyrite residues at 100°C for 2 h to heat-inactivate the biofilms of *L. ferriphilum*. And after washing, the pyrite residues were resuspended in 50 mL fresh medium which contained 0.02% yeast extracts and 10^8^ cells/mL of sulfur-grown cells of *S. thermosulfidooxidans*. Two more sets of experiment were set: (1) 5 × 10^7^ cells/mL of sulfur-grown *S. thermosulfidooxidans* cells and 5 × 10^7^ cells/mL of iron-grown *L. ferriphilum* cells were inoculated simultaneously to 2% (m/v) freshly ground sterile pyrite; (2) 10^8^ cells/mL of *S. thermosulfidooxidans* were inoculated to 2% (m/v) sterile freshly ground pyrite. All the experiments were performed at 37°C on a rotary shaker at 140 rpm with an initial pH of 1.8. Each experiment was done in triplicate.

### Adhesion Experiments

The pyrite grains with 1 day old living or inactivated biofilms of *L. ferriphilum* were prepared as described above. Then cell attachment of *S. thermosulfidooxidans* to the pyrite with living or heat-inactivated biofilms followed. Briefly, 10^8^ cells/mL of *S. thermosulfidooxidans* were inoculated into flasks filled with 50 mL MAC medium, pH 1.8, and with freshly ground sterile pyrite or with living or inactivated biofilms of *L. ferriphilum.* Then they were cultivated at 37°C with shaking at 140 rpm. The number of planktonic cells was determined by direct counting within 3 h. The amount of attached cells was calculated by subtracting the planktonic cells from the initial cell number. All the experiments were performed in triplicate.

### Bioleaching of Pyrite by *S. thermosulfidooxidans* With Leachate From *L. ferriphilum*

The culture supernatants of *L. ferriphilum* were collected after 3 or 14 days of incubation with pyrite. Then they were filtered through a filter with a pore size of 0.22 μm. Afterward, pyrite-grown cells of *S. thermosulfidooxidans* were harvested by centrifugation and after washing with fresh MAC medium they were inoculated into the filtered leachate with a final concentration of 10^8^ cells/mL. 2% (m/v) freshly ground sterile pyrite were then added to all the flasks. Considering the importance of ferric ion for leaching, two batches of experiment were set as control, in which the same amount of iron as detected in the leachate was added to the fresh medium. One control got an inoculum of *S. thermosulfidooxidans*, named as biological control, and an assay without inoculum of *S. thermosulfidooxidans* functioned as chemical control. The added iron was ferric chloride. The experiments were performed on a rotary shaker at 140 rpm, 37°C. Data were collected from three parallel experiments.

### Cell Number, pH and Iron Ion Determination

The planktonic cell density was determined by direct counting with a Thoma counting chamber (Assistant, Germany) under a light microscope (Leica DMLS, Wetzlar GmbH) in phase contrast mode with a magnification of 400 times.

To determine the attached cells on the pyrite grains, samples were stained with SYTO9 which combines with the attached cells and gives fluorescent signals. Then an epifluorescence microscope (Axio Imager A1m, Zeiss, Germany) was used to take images of the pyrite grains. Afterward, the images were analyzed by platform Fiji according to Schindelin et al. (2012) to obtain the number of attached cells. Fiji identifies and enumerates the objects in the stack with a user-defined threshold for voxel intensity value. For counting first the value of the voxel intensity threshold was set manually by visual inspection of the images (*Fiji: Image* > *Adjust* > *Threshold*). Some incomplete cells, caused by the adjustment of the threshold, were then filled by function of fill holes (*Fiji: Process* > *Binary* > *Fill Holes*). To distinguish conjunct cells the watershed function was applied (*Fiji: Process* > *Binary* > *Watershed*). Afterward, the counting process was performed (*Fiji: Analyze* > *Analyze Particles*). Each cell number was collected from 25 images.

The pH was determined with a digital pH meter (Model pH 537, WTW, inLab 422 Combination Semi-micro pH Electrode, Mettler Toledo).

Ferrous and total iron ions were quantified by the procedures described by Tamura (Tamura et al., 1974). The principle is that 1,10-Phenanthroline complexes ferrous ions. The complex gives a red color and can be measured spectrophotometrically at 492 nm. Subsequently, the total iron can be quantified by adding hydroxylamine, which reduces ferric to ferrous ions. The samples were measured in triplicate within microtiter plates with a UV-Vis spectrophotometer (TECAN, Infinite pro^®^ 200).

### AFM and Epifluorescence Microscopy (EFM) Instrumentation and Performance

A “BioMaterials Workstation” (JPK Instruments, Germany) composed of a NanoWizard II atomic force microscope (JPK Instruments) and an upright epifluorescence microscope (Axio Imager A1m, Zeiss, Germany) was applied to visualize the cells and the biofilms. A sample can be visualized at the same site between the two microscopes with 2 μm deviation (Mangold et al., 2008). In this study all samples were first stained by 6 μM SYTO9. The location was first chosen by EFM, then visualized by AFM. For AFM imaging, the contact mode was used. The applied setpoint was below 1 nN and the scan rate ranged from 0.1 to 0.5 Hz. A CSC38/NO AL (Mikromasch, Tallinn, Estonia) probe was used for imaging and cantilever B with the following parameters were chosen for scanning: length, 350 μm; width, 32.5 μm; thickness, 1.0 μm; resonance frequency, 10 kHz; shape, cone with a full cone angle of 40^*o*^; and force constant, 0.03 N/m. Before the experiments the cantilevers were immersed in Piranha solution for 10 min and then washed with sterile MilliQ water. All AFM operations were performed in MAC medium at pH 2.5 at room temperature. The sensitivity of the optical lever system and the cantilever spring constant were calibrated in liquid prior to each experiment. The spring constant for each cantilever was compatible with the manufacturer’s specifications.

## Results and Discussion

### Biofilm Formation by Pure Cultures

**FIGURE 1:**
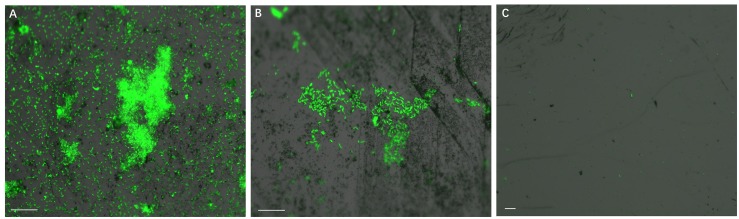
Biofilm formation on pyrite slices by a pure culture of *S. thermosulfidooxidans* under 37°C with an initial pH value of 1.8. Images **(A–C)** show 1, 7 and 16 days old biofilms of *S. thermosulfidooxidans*, respectively. Scale bar, 20 μm.

**FIGURE 2:**
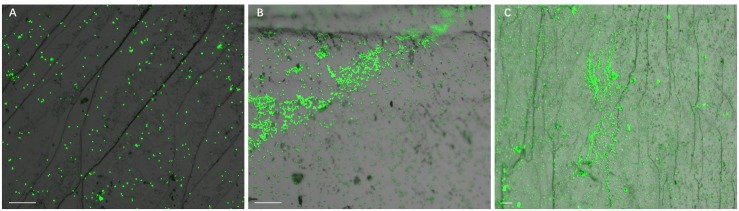
Biofilm formation on pyrite slices by a pure culture of *L. ferriphilum* under 37°C with an initial pH value of 1.8. Images **(A–C)** show 1, 7, and 16 days old biofilms of *L. ferriphilum*, respectively. Scale bar, 20 μm.

Development of biofilms on pyrite slices by a pure culture of *S. thermosulfidooxidans* or of *L. ferriphilum* within 14 days is shown in Figure 1 or Figure 2, respectively. Figure 1A clearly indicates that after 1 day incubation cells of *S. thermosulfidooxidans* had attached on the pyrite slices either in the form of single cells or flocs. However, the attached cells detached gradually and almost no cells could be observed after 16 days of incubation (Figure 1C). It has been reported that cells of *S. thermosulfidooxidans* are unable to form stable biofilms on pyrite, if a routine culturing procedure is used. The cells are non-motile, but they prefer to colonize the defect sites of mineral surfaces (Sand et al., 2001). Also the culturing environment deteriorates as the experiment proceeds (e.g., decrease of organic compounds and increase of metabolic end products), which might stimulate formation of endospore (Li et al., 2016). In the assays with *L. ferriphilum* after 1 day of incubation most cells had attached on the surface of the pyrite slices in the form of single cells (Figure 2A). The cells were observed to grow in colonies after 7 days of incubation (Figure 2B). More robust biofilms of *L. ferriphilum* were observed after 16 days of incubation (Figure 2C).

### Adhesion of Cells of *S. thermosulfidooxidans* to Pyrite in the Presence of *L. ferriphilum*

**FIGURE 3:**
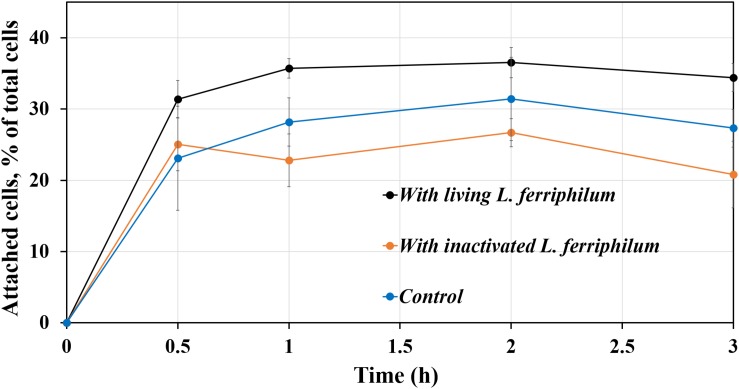
Adhesion of *S. thermosulfidooxidans* to pyrite pre-colonized by 1 day old biofilms of *L. ferriphilum* within 3 h under 37°C, pH 1.8.

Figure 3 demonstrates that the number of attached cells of *S. thermosulfidooxidans* on pyrite changes as a function of time. The pyrite was pre-colonized with 1 day old living or inactivated biofilms of *L. ferriphilum.* In the sterile control after 3 h about 27% of the inoculated cells of *S. thermosulfidooxidans* had attached on the pyrite. Compared with the sterile control, the percentage of attached cells of *S. thermosulfidooxidans* increased by around 5%, if the pyrite had been pre-colonized with living cells of *L. ferriphilum*. However, it decreased by approximately 5%, if the pyrite had been pre-colonized with inactivated cells of *L. ferriphilum*. It has been reported that bacterial adhesion is influenced by many factors, including surface roughness, type of mineral, hydrophobicity and medium (Dexter et al., 1975; Absolom et al., 1983; Helke et al., 1993; Quirynen et al., 1993; Blackman and Frank, 1996). It is likely that a conditioning film formed on the pyrite surface by excretions of the cells of *L. ferriphilum* like EPS compounds, which changed surface properties of the pyrite surfaces. This influenced the adhesion of cells of *S. thermosulfidooxidans*. However, in the assay with the inactivated biofilm of *L. ferriphilum* the conditioning film and the inactivated cells of *L. ferriphilum* might have been destroyed or deteriorated and blocked the attaching sites. Consequently, the attached number of cells of *S. thermosulfidooxidans* decreased.

### Biofilm Formation of *S. thermosulfidooxidans* in the Presence of *L. ferriphilum*

**FIGURE 4:**
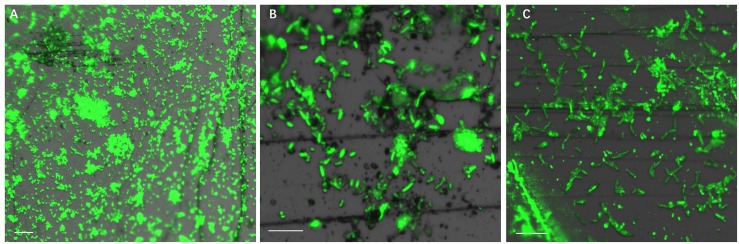
Biofilm formation by cells of *S. thermosulfidooxidans* and of *L. ferriphilum* on pyrite under 37°C with an initial pH value of 1.8. The two cells were simultaneously inoculated. Images **(A–C)** show 1, 7 and 16 days old binary biofilm of *S. thermosulfidooxidans* and *L. ferriphilum*, respectively. Scale bar, 10 μm.

**FIGURE 5:**
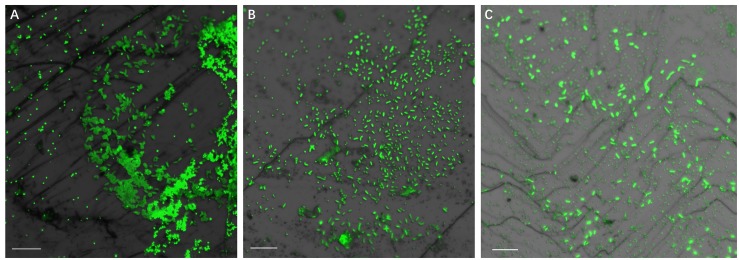
Biofilm formation by *S. thermosulfidooxidans* on pyrite pre-colonized with 1 day old living biofilms of *L. ferriphilum* under 37°C with an initial pH value of 1.8. Images **(A–C)** show the binary biofilm of *S. thermosulfidooxidans* and *L. ferriphilum* after 1, 7, and 16 days incubation. Scale bar, 10 μm.

**FIGURE 6:**
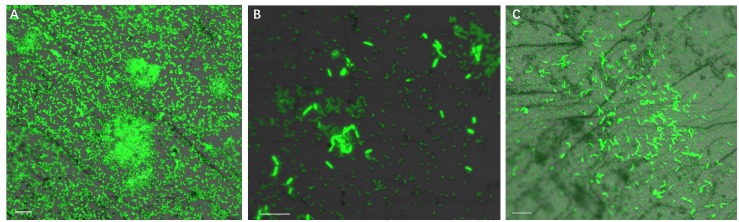
Biofilm formation by *S. thermosulfidooxidans* on pyrite pre-colonized with 1 day old inactivated biofilm of *L. ferriphilum* under 37°C with an initial pH value of 1.8. Images **(A–C)** show the binary biofilm of *S. thermosulfidooxidans* and *L. ferriphilum* after 1, 7 and 16 days incubation. Scale bar, 10 μm.

**FIGURE 7:**
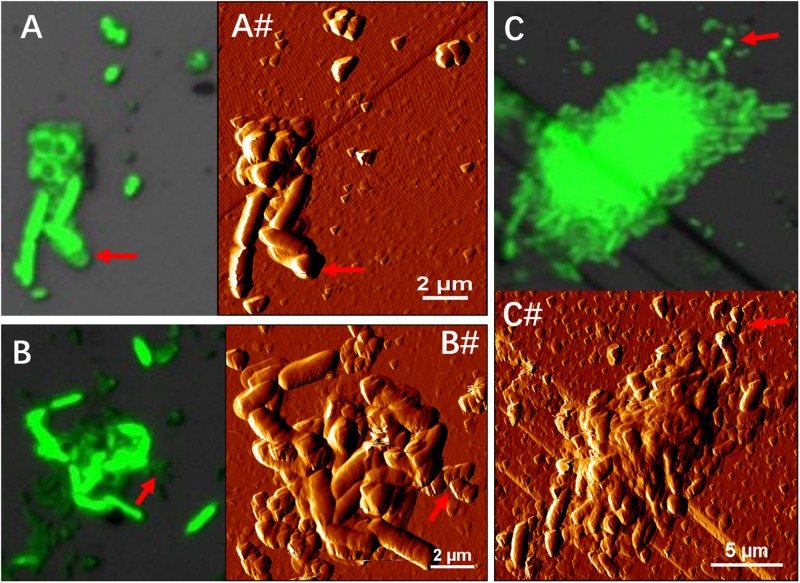
EFM **(A–C)** combined AFM **(A#–C#)** images indicate physical contacts between cells of *S. thermosulfidooxidans* and cells of *L. ferriphilum* during biofilm formation on pyrite under 37°C with an initial pH value of 1.8. Images **(A,A#,B,B#)** show the biofilms of *S. thermosulfidooxidans* on pyrite pre-colonized with 1 day old living and inactivated biofilms of *L. ferriphilum*. Images C and C# show biofilm developed by culture of *S. thermosulfidooxidans* and *L. ferriphilum* on a clean pyrite. All the red arrows in images indicate the cells of *L. ferriphilum*.

Figure 4 illustrates biofilm formation on sterile pyrite by a binary culture of *S. thermosulfidooxidans* with *L. ferriphilum* within 16 days. The two species were simultaneously inoculated. Figures 5, 6 show the biofilm formation by the culture of *S. thermosulfidooxidans* on pyrite pre-colonized with living or inactivated biofilms of *L. ferriphilum*, respectively. It is visible that the cells of *S. thermosulfidooxidans* are long rod-shaped, while the cells of *L. ferriphilum* are vibrioid and/or round-shaped and small. The two cell forms can be distinguished well with the microscope. In all three assays after 16 days incubation the biofilms of *S. thermosulfidooxidans* remained on the pyrite. It indicates that *S. thermosulfidooxidans* can form biofilms on pyrite in the presence of *L. ferriphilum*. However, the architecture of the biofilm is different. Biofilms of *S. thermosulfidooxidans* in the form of single cells are observed on the pyrite pre-colonized with living biofilms of *L. ferriphilum* (Figure 5). In the other two assays biofilms of *S. thermosulfidooxidans* grow in the form of colonies with biofilms of *L. ferriphilum* (Figures 4, 6). In all assays a physical contact between the cells of *S. thermosulfidooxidans* and *L. ferriphilum* can be observed, as is visible in the images of EFM and AFM in Figure 7.

A physical contact has been reported also for other microorganisms and it happens during microbial coaggregation. Coaggregation, the process of adhesion between genetically distinct bacterial partners, was first found between bacteria isolated from human dental plaque in 1970 (Gibbons and Nygaard, 1970). It is an integral process in the development of multispecies biofilms and is caused by specific ligand receptor interactions (Rickard et al., 2003). In a dual-species coaggregation one of the species will benefit from being within a coaggregation, while the other may also gain or not gain advantages as compared to planktonic cells. Katharios-Lanwermeyer et al. (2014) reviewed coaggregation mediated interactions and concluded five potential outcomes for biofilm communities: (1) The interspecies can rapidly form aggregates because of the enhanced cell-cell adhesion; (2) Species within the coaggregation can exchange of metabolites; (3) The coaggregation can aid one species to kill/inactivate another or to protect both species from predation; (4) The coaggregation reduces the distance between cells, thereby facilitating cell-cell signaling; (5) The coaggregation can protect cells from adverse events, enhance their antimicrobial resistance and genetic exchange.

In this study, obviously, coaggregation enhanced the adhesion of *S. thermosulfidooxidans*. However, how the two species developed the coaggregation or whether it was also caused by specific ligand receptor interactions, and whether this coaggregation could facilitate metabolite exchange or cell-cell signaling remains unclear. More delicate experiments need to be done to answer these questions.

### Bioleaching of Pyrite by *S. thermosulfidooxidans* in the Presence of *L. ferriphilum*

For an improved understanding the assays are numbered as follows: (1) *S. thermosulfidooxidans* incubated with pyrite pre-colonized with 1 day old living biofilms of *L. ferriphilum*; (2) *S. thermosulfidooxidans* incubated with pyrite pre-colonized with 1 day old inactivated biofilms of *L. ferriphilum*; (3) *S. thermosulfidooxidans* and *L. ferriphilum* were simultaneously inoculated into assays with sterile pyrite; (4) pyrite colonized with 1 day old biofilms of *L. ferriphilum* (without planktonic cells); (5) *S. thermosulfidooxidans* incubated with sterile pyrite. Data for an assay with sterile pyrite are not shown, since no dissolution was detected.

**FIGURE 8:**
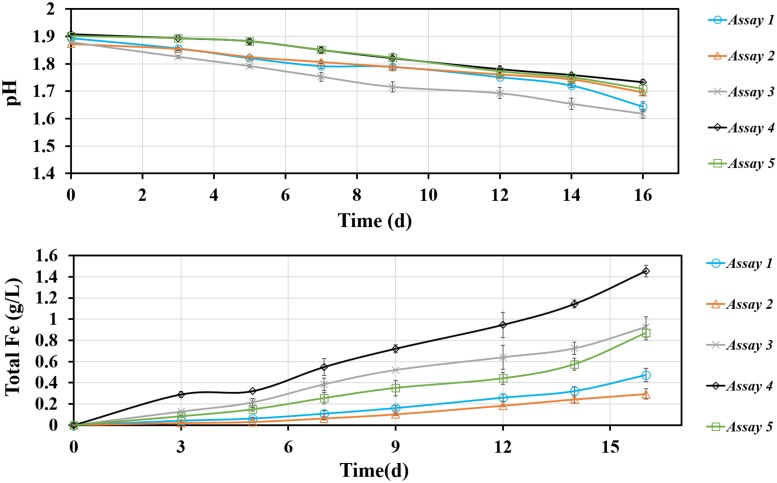
Changes of pH and total iron concentration as a function of time during pyrite bioleaching under 37°C with an initial pH value of 1.8. Assay 1, *S. thermosulfidooxidans* with 1 day old living biofilms of *L. ferriphilum* incubated in sterile medium; Assay 2, *S. thermosulfidooxidans* with 1 day old inactivated biofilms of *L. ferrriphilum* incubated in sterile medium; Assay 3, *S. thermosulfidooxidans* and *L*. *ferriphilum* were simultaneously inoculated and incubated with sterile pyrite and medium; Assay 4, Pyrite colonize with 1 day old biofilms of *L. ferriphilum* incubated in sterile medium; Assay 5, *S. thermosulfidooxidans* incubated in sterile medium with sterile pyrite.

The leaching of pyrite by *S. thermosulfidooxidans* in the presence of *L. ferriphilum* was calculated and the results are shown in Figure 8. In all assays the pH decreased, which indicates that bioleaching occurred. Since *L. ferriphilum* can oxidize only ferrous ion for energy but *S. thermosulfidooxidans* can use both, ferrous ion and RISCs, as energy source, the pH in assay 4 decreased the least. The pH decreased the most in assays 1 and 3, but in the first 14 days the pH in assay 3 was lower than it in assay 1. After 16 days of leaching the highest iron concentration was detected in assay 4 with a final value of 1.5 g/L, followed by the one in assay 3 with 1 g/L. There were 0.5 g/L and 0.3 g/L iron detected in assay 1 and assay 2, respectively. The data indicate that with biofilms of *L. ferriphilum* at the beginning of leaching, no matter whether living or inactivated, these biofilms exerted a negative effect on leaching by *S. thermosulfidooxidans*.

It is known that leaching bacteria prefer to colonize the crystal defect sites on a mineral surface and only attached cells can initiate bioleaching (Bellenberg et al., 2015). Obviously the inactivated biofilms of *L. ferriphilum* in assay 2 (Figure 8) could not perform leaching and to some extent they protected the pyrite surfaces from being attacked by ferric ions. Also the sites for attachment of *S. thermosulfidooxidans* were blocked. Consequently, the leaching needed longer time to start and the amount of leached iron was the least among all assays (Figure 8). For living biofilms of *L. ferriphilum* the introduction of *S. thermosulfidooxidans* might mean introducing a competitor for iron, which can be indicated by the higher iron concentration in assays 4 and 5 (single culture) than in assay 1. Interestingly, also with living *L. ferriphilum* the pH in assay 3 was much lower and the concentration of iron ion was much higher than in assay 1 (Figure 8). An increased concentration of iron ions mean dissolution of pyrite and a decreased pH means oxidation of RISCs. *S. thermosulfidooxidans* is the only sulfur-oxidizer in the system. Thus, most likely in assay 1 *S. thermosulfidooxidans* grew mainly on ferrous ion and the competition led to the bad leaching performance. In assay 3 the decreased pH illustrated the oxidation of RISCs by *S. thermosulfidooxidans*, but their worse leaching performance than that of the pure culture of *L. ferriphilum* indicated a negative effect on growth of *L. ferriphilum*. A possible explanation is that in assay 3 *S. thermosulfidooxidans* grew on both ferrous ion and RISCs.

Based on the results above two points can be highlighted:

1. The living state of *L. ferriphilum* at the start has a different influence on leaching performance of the binary culture and this difference might be relate to the metabolic preference of *S. thermosulfidooxidans*.
2. Contact leaching is important. Inactivated biofilms of *L. ferriphilum* blocked the attachment sites for *S. thermosulfidooxidans* on pyrite surfaces, resulting in the lowest leaching efficiency.

### Bioleaching of Pyrite by *S. thermosulfidooxidans* in Spent Leach Liquor

**FIGURE 9:**
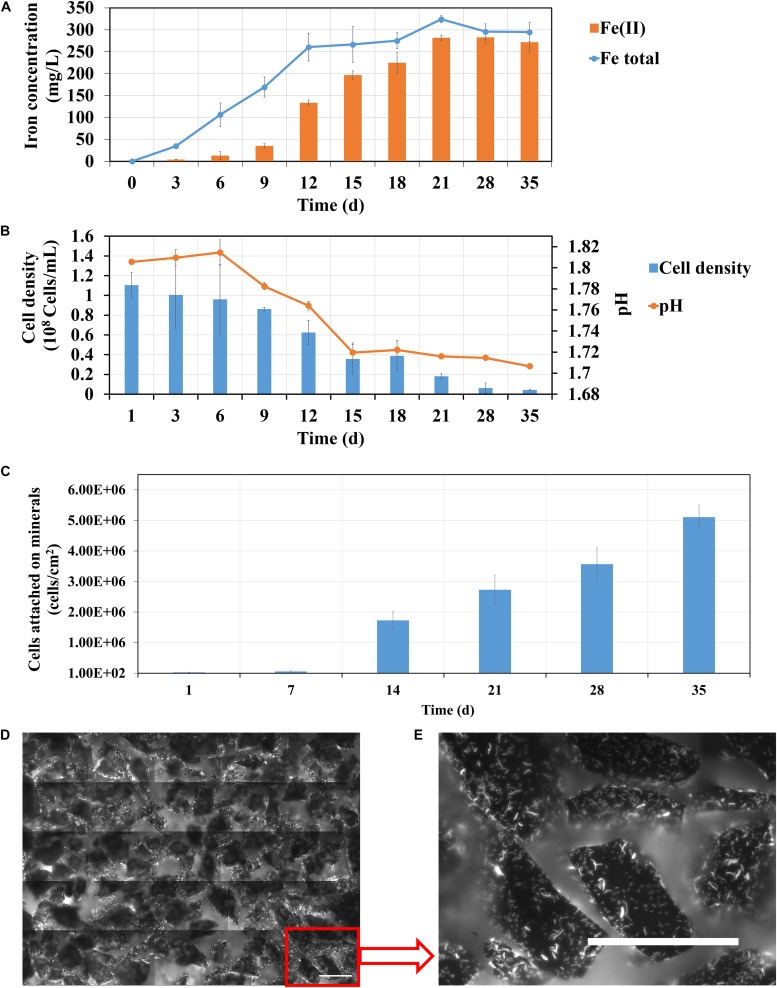
Changes of iron concentration **(A)**, cell density **(B)** and pH **(C)** during pyrite leaching by *S. thermosulfidooxidans* in basal salt medium without yeast extract addition under 37°C with an initial pH value of 1.8. **(D,E)** are EFM images showing the biofilms formed on pyrite after 35 days of leaching. Scale bar, 100 μm.

The effects of spent leach liquor from *L. ferriphilum* on pyrite leaching by *S. thermosulfidooxidans* was also investigated. In order to reproduce a natural leaching system yeast extract was not added in this section. Firstly, bioleaching of pyrite by *S. thermosulfidooxidans* without addition of yeast extract was tested and the results are shown in Figure 9. From Figure 9A it can be seen that in the first 12 days the iron concentration increased from 0 to 260 mg/L. Afterward it almost leveled off at 300 mg/L. The iron ions were detected mostly in the form of Fe(III) in the first 9 days. However, from 12 days on Fe(II) became gradually the dominant form. At the end of the experiment the iron ions were almost all in the form of Fe(II). Data in Figure 9B indicate that the pH of the leaching system decreased rapidly from the 6th day on till the 15th day when it did not change any more. The numbers of planktonic cells decreased, from the initial cell density of 1.1 × 10^8^ cells/mL to the final cell density of 4 × 10^6^ cells/mL. The number of attached cells was also monitored. The bar chart (Figure 9C) indicates that the number of attached cells increased during the leaching and, finally, the pyrite surfaces were covered with 5 × 10^6^ cells/cm^2^ (Figures 9D,E). Our data clearly indicate that after around 14 days of incubation the bioleaching stopped and the cells of *S. thermosulfidooxidans* started to adhere to pyrite surfaces. It remains, however, unclear, why these events occurred. A similar phenomenon has been reported, where adhesion of *Acidithiobacillus ferrooxidans* to pyrite was enhanced if no phosphate was added to the growth medium (Bellenberg et al., 2012). In our study lack of nutrition, such as yeast extract, might account for the observed events.

**FIGURE 10:**
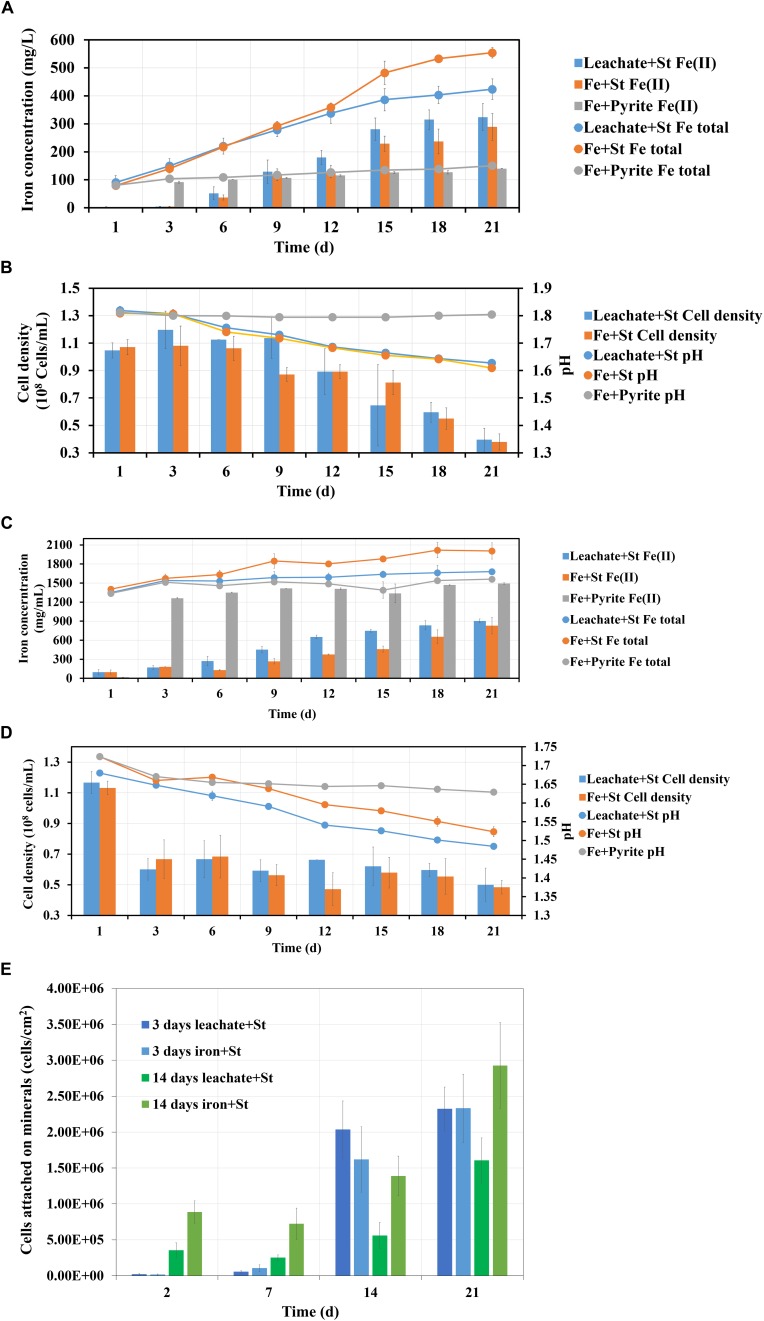
Images **(A–E)** demonstrate the changes of iron concentration, cell density and pH during pyrite leaching by *S. thermosulfidooxidans* with 3 days **(A,B)** and 14 days **(C,D)** pyrite leachate collected from *L. ferriphilum* under 37°C. Image **(E)** shows the number of attached cells of *S. thermosulfidooxidans* on pyrite. Leachate + St, *S. thermosulfidooxidans* performs pyrite leaching in pyrite leachate collected from *L. ferriphilum*; Fe + St, *S. thermosulfidooxidans* performs pyrite leaching in MAC medium with initial addition of same amount of iron detected in the pyrite leachate collected from *L. ferriphilum*; Fe + pyrite, pyrite leaching in MAC medium only with initial addition of same amount of iron detected in the pyrite leachate collected from *L. ferriphilum*.

Bioleaching of pyrite by *S. thermosulfidooxidans* with pyrite leachate from *L. ferriphilum* was then investigated. Figure 10 shows, how each parameter changes during bioleaching, if the bioleaching was performed in 3 days or 14 days old pyrite leachate from *L. ferriphilum*. Although the leachates were of different age, the results of the three assays were similar: (1) The bioleaching ran better in the biological control than with the leachate; (2) A large amount of Fe(II) was detected in all leaching assays at the end of the experiment (Figures 10A,C); (3) Except for the chemical control the pH in all leaching assays decreased and the planktonic cell densities of *S. thermosulfidooxidans* also decreased (Figures 10B,D); (4) The number of attached cells increased in all leaching assays (Figure 10E).

There are also differences: (1) The age of the leachate influenced the time until the iron concentration started to differ. Specifically, if *S. thermosulfidooxidans* was incubated with 3 days old leachate, only after 12 days the total iron concentration became lower than in the biological control (Figure 10A). If the cells were incubated in 14 days old leachate, the concentration of total iron differed between the test assays and the biological control from the 3rd day on (Figure 10C). Also due to the age of the leachate the percentage of Fe(II) was different. With 3 days old leachate after 21 days of bioleaching the Fe(II) concentration amounted to 76%. In the biological control the percentage of Fe(II) was 52% (Figure 10A). If the leachate was 14 days old, the assays after 21 days contained 53% of Fe(II), whereas 41% of Fe(II) was measured in the biological control (Figure 10C). (2) The time, until a decrease of the pH occurred, differed also. In the assays with 3 days old leachate the pH started to decrease from the 3rd day on in both assays (Figure 10B). In the assays with 14 days old leachate the pH in both groups decreased right from the start (Figure 10D). (3) For adhesion differences were noted. In the assays with 3 days old leachate in all assays from the 14th day on the cells were detected mostly attached to pyrite surfaces, while in the assays with 14 days old leachate from the very beginning the cells of both assays were detected largely attached on pyrite surfaces. However, in the assays with 14 days old leachate the numbers of attached cells were always lower than it in the biological control, which should be related to the organic products produced by *L. ferriphilum* (Figure 10E).

**FIGURE 11:**
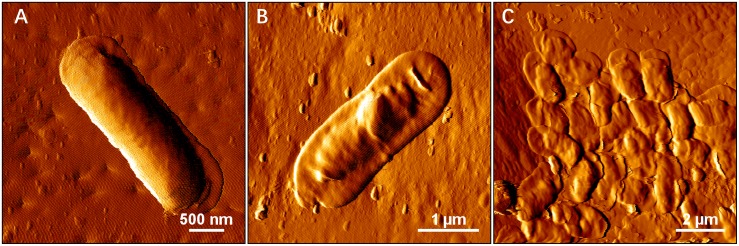
AFM images show biofilm formation on sulfur slices by *S. thermosulfidooxidans*. Images **(A–C)** show biofilms after 4 h, 2 and 5 days incubation with sulfur slices in MAC medium with initially addition of 0.02% yeast extracts, respectively.

From these results three points can be concluded:

1. Autotrophic (control) or rare organic (leachate) nutrition would lead *S. thermosulfidooxidans* to adhere on pyrite after a certain time of bioleaching, but the adhesion does not show positive effects on bioleaching. With the adhesion a large amount of Fe(II) is detected in the leaching system.
2. Iron in the bioleaching system at the very beginning would affect the bioleaching by *S. thermosulfidooxidans* under autotrophic or rare organic nutrition conditions: when there is no iron, the bioleaching would level off after a certain time (Figure 9); however, when there is iron, the bioleaching would continue or at least the oxidization of RISCs would continue (Figure 10). Most likely biofilms of *S. thermosulfidooxidans* grow mainly on RISCs (if enough exist) especially under the condition of rare organic nutrition and/or of high concentration of iron ion. Before we cultivated *S. thermosulfidooxidans* with sulfur slices and we found that they could develop biofilms although the yeast extract in medium was consumed as time went by (Figure 11). However, it could not develop biofilms on pyrite slices (Figure 1).
3. Based on differences 1 and 3 described above, it can be concluded that the leachate from *L. ferriphilum* affects iron oxidization and adhesion of *S. thermosulfidooxidans* (Figure 10).

## Conclusion

Adhesion to and biofilm formation on mineral surfaces are critical steps in bioleaching. Understanding the interactions between two important species is helpful to design a microbial community for bioleaching or to prevent AMD. In this study we checked the effects from *L. ferriphilum*, including its planktonic cells, its living biofilms, its inactivated biofilms or its metabolic substances, on bioleaching of pyrite by *S. thermosulfidooxidans*. Our studies indicate the importance of contact leaching. Coaggregation of *L*. *ferriphilum* and *S. thermosulfidooxidans* can enhance the biofilm formation by *S. thermosulfidooxidans* on pyrite surfaces. However, the way of addition of the two species shows different effects on bioleaching, which might be related to metabolic preference of *S. thermosulfidooxidans*. If the minerals are pre-colonized with biofilms of *L. ferriphilum*, *S. thermosulfidooxidans* prefers to grow on iron. Consequently, a competition between the two species lead to a worse leaching performance than that of either pure culture. However, if the cultures of *S. thermosulfidooxidans* and of *L. ferriphilum* are simultaneously inoculated to a leaching system, *S. thermosulfidooxidans* grows on both iron and RISCs, resulting in an improved leaching efficiency compared with the leaching performed by pure *S. thermosulfidooxidans*. In summary the leaching performance can be ranked as follows: biofilms of *L. ferriphilum* (Lf) > mixtures of Lf + *S. thermosulfidooxidans* (St) > St > St + living biofilms of Lf > St + inactivated biofilms of Lf. Thus, the role of *S. thermosulfidooxidans* in a short-term leaching system is most likely decided by the time/sequence of introduction into the habitat.

## Data Availability Statement

All datasets generated for this study are included in the article/supplementary material.

## Author Contributions

QL, TX, and WS designed the experiments. QL, JZ, SL, and RZ performed the experiments and analyzed the data. QL, TX, and WS prepared the manuscript. All authors read and approved the final manuscript.

## Conflict of Interest

The authors declare that the research was conducted in the absence of any commercial or financial relationships that could be construed as a potential conflict of interest.
